# Supplementary material for: Orthogonal navigation of multiple visible-light-driven artificial microswimmers
Source: Nat Commun. 2017 Nov 10;8:1438. doi: 10.1038/s41467-017-01778-9 (PMC5681650; doi:10.1038/s41467-017-01778-9)
Supplement: Supplementary file 1 — Supplementary Information [file 41467_2017_1778_MOESM1_ESM.pdf]

Parameters	Meaning	Value
D_1	H^+ diffusion coefficient	9.31×10^{-5} [$cm^2\cdot s^{-1}$]
D_2	OH^- diffusion coefficient	5.03×10^{-5} [$cm^2\cdot s^{-1}$]
D_3	H_2O Diffusion coefficient	2.26×10^{-5} [$cm^2\cdot s^{-1}$]
F_c	Cathode OH^- flux	1.90×10^{-3} [$mol\cdot m^{-2}\cdot s^{-1}$]
F_a	Anode H^+ flux	6.25×10^{-3} [$mol\cdot m^{-2}\cdot s^{-1}$]
$L_{\mathrm{T_iO_2}}$	Length of TiO_2 head	4 [μm]
$d_{\mathrm{T_iO_2}}$	Diameter of TiO_2 head	3 [μm]
$L_{\mathrm{S_i}}$	Length of Si trunk	6 [μm]
$d_{\mathrm{S_i}}$	Diameter of Si trunk	0.8 [μm]

Supplementary Table 1. Typical parameters used in the simulation.

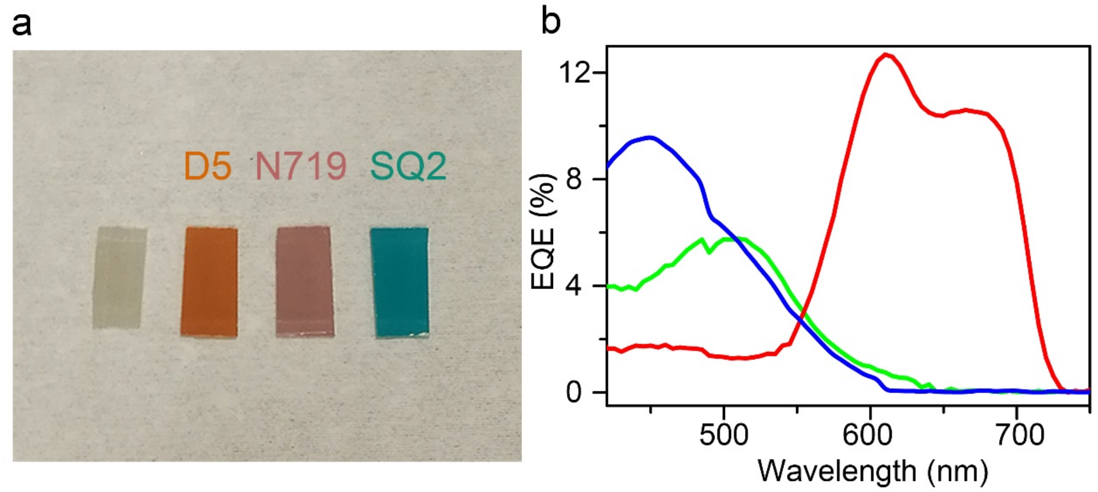

Supplementary Figure 1. a. The photograph of pristine and dye-sensitized TiO_2/FTO samples. **b.** The external quantum efficiency (EQE) of the nanowire-based dye-sensitized solar cells (D5 (blue), N719 (green) and SQ2 (red)).

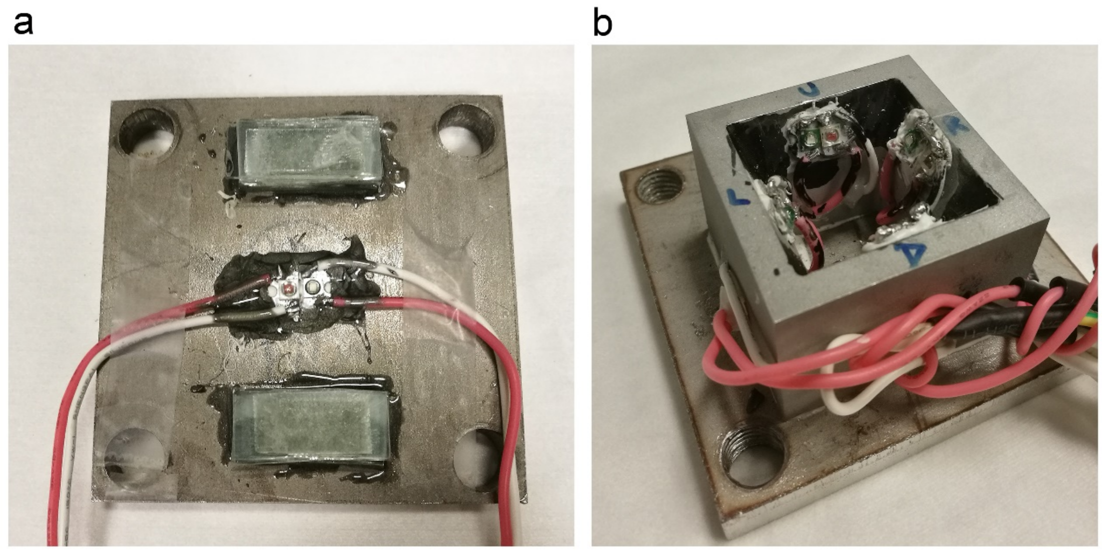

Supplementary Figure 2. a. The customized stage for intensity-dependent speed measurement and multi-channels light manipulation. **b.** Stage with independently controllable red and blue LEDs over orthogonal directions.

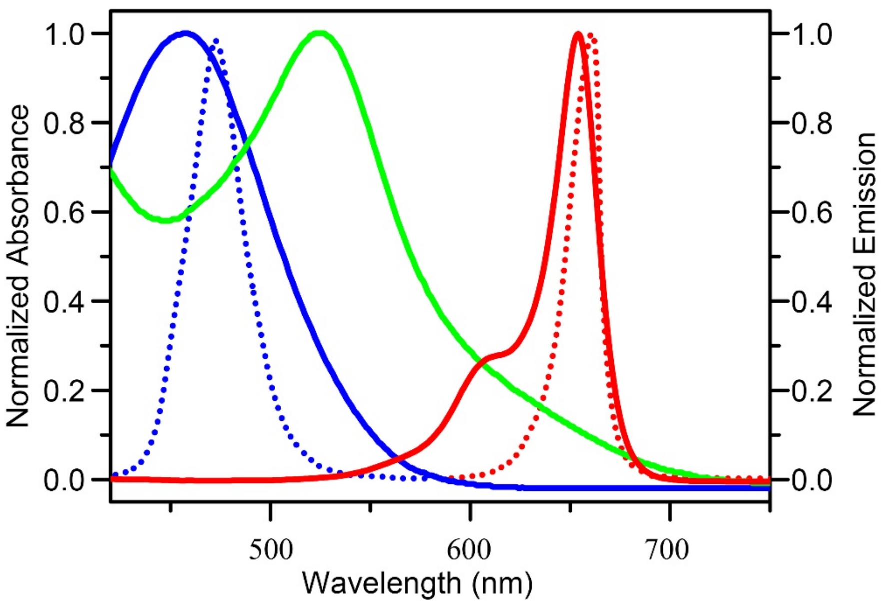

Supplementary Figure 3. The normalized absorbance of ethanolic solution of dye (D5 (blue), N719 (green) and SQ2 (red)). The dotted curve indicated the normalized emission spectra of LED (475 nm (blue), 660 nm (red)).
