# Supplementary material for: Orthogonal navigation of multiple visible-light-driven artificial microswimmers
Source: Nat Commun. 2017 Nov 10;8:1438. doi: 10.1038/s41467-017-01778-9 (PMC5681650; doi:10.1038/s41467-017-01778-9)
Supplement: Supplementary file 3 — Description of Additional Supplementary Files [file 41467_2017_1778_MOESM3_ESM.pdf]

Description of Additional Supplementary Files

File Name: Supplementary Movie 1
Description: Demonstration of multi-channel light manipulating D5 and SQ2 sensitized microswimmers with alternating 475 nm (Blue) and 660 nm (Red) light illumination. The movie is accelerated by three times.

File Name: Supplementary Movie 2
Description: The rotation demonstration of D5 and SQ2 sensitized microswimmers under 475 nm (Blue) and 660 nm (Red) LED light respectively. The movie is accelerated by five times.

File Name: Supplementary Movie 3
Description: The spontaneous controlling of two light-guided microswimmers to spell ‘r’ and ‘b’. The movie is accelerated by ten times.
